# Hospital Meetings

**Published:** 1896-03-07

**Authors:** 


### THE INSTITUTIONAL WORKSHOP.

### HOSPITAL MEETINGS.

### EAST LONDON HOSPITAL FOR CHILDREN, SHADWELL.

Re-opening of the Wards.

In spite of a dull day and a bitter east wind quite a number of visitors found their way to Shadwell last week on the occasion of the formal re-opening of the Hospital for Children. The Duchess of Albany arrived at three o'clock, and was first shown over the wards by the members of the Reception Committee, amongst whom were the Earl and Countess of Strafford, the Earl and Countess of Erroll, Mr. Trinder (chairman of the board of management), Cardinal Vaughan, the Chief Rabbi, Mr. Cheston, Mr. H. C. Burdett, and others. Afterwards Her Royal Highness was conducted to the large out-patient waiting hall, where the guests were assembled, and an address of welcome was read by Lord Strafford, thanking her for her presence, and enumerating the various improvements effected in the building. These were stated to include new bath-rooms at the entrance to the wards, enabling patients to be bathed before entering the wards; new lavatories and ward offices in a separate annexe, cut off by cross ventilation; improved heating and ventilation, and a general overhauling of the drains; besides the addition to the floor and cubic air space by the removal of a partitioned room from each ward formerly used as the sister's bed-room. These works were superintended by Mr. Horace Cheston, with the advice of Dr. Dawson Williams in sanitary matters. The expenses entailed by the alterations amounted to about £3,000, of which sum £1,500 was still uncollected. Founded by Dr. Heckford with only ten beds in 1868, the hospital now contains 102, and treats a large number of out-patients, most of them coming from crowded and miserably poor homes.

The Duchess of Albany having briefly declared the wards re-opened,

Lord Strafford proposed a vote of thanks to Her Royal Highness, and, in doing so, spoke of the kindly interest ever shown by the Royal Family in charitable undertakings.

A fact of some historical interest was mentioned by Mr. Trinder, who pointed out that, oddly enough, two ancient families who had held property in Shadwell and Stepney since the days of Charles II.—the Wentworths and the Cromwells—were still represented on the governing body of the Shadwell Hospital, the former by its president (Lord Strafford).

Offerings of purses were presently made to the Duchess, several of them by little patients, carried on to the platform by the nurses, and looking their very best in pretty white pinafores and red frocks and ribbons.

A vote of thanks was proposed to the visitors, and, in replying, both Cardinal Vaughan and the Chief Rabbi testified of their own personal knowledge to the good work done by this hospital, situated, as it was, in a part of London which, perhaps more than any other, needed its ministrations. The kindness and care of the medical and nursing staffs had earned the confidence and gratitude of all concerned in the hospital and its work. The Chief Rabbi caused some amusement by remarking that he thought a certain military command should be heard with another and a special significance within those walls—"Present arms" might be translated into "Present alms to this hospital.'

Mr. Burdett, from whom, as "one whose business and pleasure it was to look into hospital matters, and who had taken the deepest interest in the East London Hospital," a few words were especially requested by Mr. Trinder, said it was a peculiar pleasure to him to be present that day, not only because of his interest in hospitals (and it had been said of him that if "Calais" were the word written on Queen Mary's heart, "hospital" would be that found on his), but because he could well remember, more than twenty years ago, the simple sail loft in which Dr. Heckford had established his ten cots. He was a good and noble man, but for whose efforts they would not that day be assembled in that splendid hospital, for splendid hospital it was. The floors, which would have been unattainable in Dr. Heckford's day, would have rejoiced his heart. They did credit to the management, and to the nurses and the officials, as did also the perfect order and cleanliness which reigned behind the scenes as in the wards. He felt it was a great honour to have the Duchess of Albany amongst them that day, and he hoped the service she had thus rendered to the hospital would inspire many others to give in money or personal service, for it was in the power of everyone to give the one or the other. The contributors really deserved no thanks at all. It was a splendid thing to have a good object ready when a man had five pounds in his pocket to spend in charity, and the thanks were rather due to the management and officers of the hospital for the excellent way in which the money given had been expended.

The wards, which were afterwards visited by the guests, looked charming with their polished floors and coloured windows, and were gay with flowers, whose tasteful arrangement, as well as the whole aspect of the hospital, did the utmost credit to the nurses. And, apart from the wards, there were other more practical attractions, which won much admiration from those who understood their significance. The new bath-rooms are delightful with their porcelain baths and fittings, and an excellent plan has been here followed in keeping each child's towel separate, distinguished by a number corresponding to the bed, and all hung on a neat rail round the walls. A special lift for conveying certain of the soiled linen straight away from the ward has been cleverly arranged outside the ward annexes, depositing it in large baskets below, from whence it is taken straight to the laundry.

### MIDDLESEX HOSPITAL.

The Annual General Court of Governors of the Middlesex Hospital was held on Thursday, February 27th, Sir Lionel McSwinnerton Pilkington in the chair. The governors usually muster in force on these occasions, and this year's meeting was no exception to the rule. Amongst those present were the Right Hon. Sir Ralph Thompson, K.C.B. (chairman of the weekly board), Mr. Francis Hoare and Mr. Marjoribanks (treasurers), Sir Arthur Townley Watson, Sir Francis Grenfell, the Rev. Canon Acheson, the Hon. C. B. Lyttelton, Mr. B. Bancroft, Colonel the Hon. C. G. Gathorne Hardy, Lieut.-Colonel J. Lambert Mears, Dr. Cayley, and Mr. Leopold Hudson. The report, the adoption of which was moved by Sir Ralph Thompson, was a particularly interesting and satisfactory one, showing that the Middlesex Hospital is carrying on successfully a great work, and that that work continues to receive much help and encouragement from the public. The fête which was to have taken place in 1895 to mark the one hundred and fiftieth birthday of the hospital, and was unavoidably postponed, is announced for July 1st next, when the Duke and Duchess of York have consented to be present. It will be held in the buildings and grounds of the hospital. A very large increase took place in the number of patients treated during 1895; these, in all departments, numbered 46,326, an increase of more than 4,000 over the number for the previous year. The great bulk of this increase is, of course, traced to the casualty and out-patient department. The year's income of £32,540 6s. 10d. showed an increase of £2,880 8s. 1d. over that for 1894, the increase being entirely in the income derived from ordinary sources. Donations both to the general account and to the cancer fund, showed an increase. There was also a slight growth in the sum received from the alms boxes. Several substantial legacies fell to the share of the Middlesex Hospital during the year, and the Hospital Sunday Fund grant was considerably in excess of that for last year. In connection with this grant the report specially drew attention to the fact that all thanks were due from everyone interested in hospital work in London for the special efforts made during the past year which had resulted in raising the total collection on behalf of the Sunday Fund to a sum never before reached. The new convalescent home at Clacton-on-Sea is approaching completion, and will, it is hoped, be ready for occupation by July 1st. The new operating theatre was an immense improvement carried through during the past year, no pains having been spared to make it as complete as modern requirements demand. Contributions towards the new cancer wing, the next undertaking to be carried through, already amount to £9,300, £12,000 having been the original estimate. Under the head of administration the report pointed out that "the cost of management showed a reduction of £64 14s. 3d., bearing a percentage of 4·6 to the ordinary expenditure, and of 3·7 to the total expenditure of the year, as compared with percentages of 4·9 and 4·8 respectively in the year preceding." The adoption of the report was seconded by Mr. Griffith Jarrett, and unanimously passed, after which the court proceeded with the re-election of officers, and other formal business.

### ROYAL FREE HOSPITAL.

Mr. Justice Bruce presided over the sixty-eighth annual court of governors held on February 26th at the Royal Free Hospital. A satisfactory report of the year's work was presented. The important event of the year had been the opening of the new front building by the Prince and Princess of Wales. The report stated that 150 students of the London School of Medicine for Women were attending the hospital and school during the year, and 25 had qualified as medical practitioners. Several of these ladies had greatly distinguished themselves at the various examinations; Miss Aldrich-Blake obtained the diploma of M.S. Lond., being the first woman to secure this degree; five former students obtained the M.D., seven the M.B., and five the B.S. degree of the London University. The work of the nursing department, under Miss Wedgwood's management, had been thoroughly efficient; the nurses did themselves and their hospital great credit at their annual examination. Several of the sisters and nurses had availed themselves of the arrangements made with the Royal National Pension Fund, whereby half their premiums are defrayed by the hospital.

Mr. Justice Bruce, in moving the adoption of the report, commented upon the completion of the new building, and hoped that the remaining debt of £1,676 might speedily be cleared off. The regular income fell short of the regular expenditure, and he trusted every effort would be made to increase the annual subscriptions. After the meeting a marble memorial of Mr. George Moore, chairman of committee from 1858 to 1864, was unveiled in the ward dedicated to his memory. Mr. Moore's services to the hospital were many, said the Chairman, and his name should ever be associated with the institution to whose benefit he gave not only money, but the most whole-hearted personal devotion for many years. The cost of the memorial has been defrayed by Mr. W. Parkin Moore, of Whitehall, Cumberland, the sculptor being Mr. Adams Acton. The tablet is a replica of one in Carlisle Cathedral, executed by the same artist, and bears the inscription, "George Moore, whose valuable services to the Royal Free Hospital are gratefully remembered. Died Nov. 21, 1876."

### GREAT NORTHERN CENTRAL HOSPITAL.

The annual meeting of the General Council of Governors of the Great Northern Central Hospital was held in the board-room of the hospital on the 28th ult., Mr. C. T. Murdoch in the chair.

The report and accounts having been taken as read,

The Chairman moved their adoption. The past year had been, he said, not only one of considerable importance to the hospital, but one of steady progress in usefulness, and had, he hoped, also tended to increase its popularity in the district and in London generally. It had been found that it would be a matter of very great assistance to the hospital if a convalescent home could be established in connection with it. During the past year there had been a decrease in the number of out-patients. This decrease had been caused, undoubtedly, by the strict system of inquiry into all cases, which had now been in force for some time. Although there had been a decrease in the actual number of patients, yet there had been an increase in the number of attendances, showing that the cases which had been received in the out-patient department had been of a more serious character, and, further, were cases which it was a real charity to relieve. Another point brought out by the report and accounts was this. They had always wished that the hospital should be to a very great extent a local one, and it was therefore a little disappointing to find that the number of subscribers resident in the district which the hospital served was so small. He could not help thinking that that matter partly arose from the fact that there were many people in the district who did not think that small donations and small subscriptions were of much value. There could not be a greater mistake than this. If only those who were in very moderate circumstances would make small donations or small subscriptions to the hospital, not only would the amount received in the aggregate be very considerable, but the interest in the hospital would be very largely increased. To the Ladies' Association, who during the past year had been very much to the fore, he tendered on behalf of the governors and committee of management their most sincere and hearty thanks. A matter of very great satisfaction was the fact that Her Royal Highness the Duchess of York took such an interest in the hospital. He did not doubt that they had very good friends at Court who had been instrumental in drawing her attention to that charity, and they were deeply thankful to Her Royal Highness for the interest she had taken in the past and was taking at the present time; and that interest, it must be remembered, was not only, as it were, the interest that the Royal Family took in all matters of this kind, but it was a personal interest. With regard to finance he wished to point out that there had been 107 beds in occupation during 1895, as against 90 in the year 1894. Naturally that additional number of beds had caused an additional amount of expenditure. The number of in-patients too had increased by 220. The additional cost of this increase in beds and patients during the year had been £1,465. Therefore the additional 220 patients had cost the hospital on an average only £6 13s. 2d. each. That showed that not only was the working of the hospital very economical, but, what was more, they had a staff at their disposal which would enable them to increase the number of beds if only the income was provided to enable them to do so. Indeed all the beds in the hospital could be filled at very little extra cost as far as management was concerned. Then, too, the total income for 1895 showed a considerable increase. It amounted to £11,288, as against an expenditure of £9,623. It had been necessary during the past year to invest as much as £4,388 for the endowment fund, which made their balance over £2,000 on the wrong side.

Mr. Alfred Royle and Mr. C. T. Bartley both emphasised the pressing need for more annual subscriptions, even though of small amount.

Mr. W. BURDETT-COUTTS said that the paragraph in the report which mentioned the work of the Ladies' Association did not go quite far enough. It was there stated that that association had raised £11,000 for the building fund, whereas the total sum it had raised for the hospital was no less than £13,450.

Dr. GLOVER remarked that people were proud of hospitals, but not of the out-patient department. He found on inquiry that at the Great Northern Central Hospital the out-patients bore a far larger proportion to the in-patients than they did in almost any other hospital in London. He was sure no one would maintain that theirs was a poor neighbourhood in the sense that the East-end was poor, and yet they had a comparatively larger number of out-patients than even the London Hospital.

The honorary treasurer and honorary secretary having been re-elected,

On the motion of Mr. FRASER BLACK, seconded by Mr. W. HARKNESS, resolutions limiting the privilege of naming a bed to the life of donors of one hundred guineas, and in the case of corporations to 15 years, were passed.

The meeting then terminated with a vote of thanks to the chairman.

### SEAMEN'S HOSPITAL SOCIETY.—ANNUAL COURT.

On February 28th, at noon, the seventy-fifth annual court of the Seamen's Hospital Society was held at the Mansion House; there was a good attendance. The chair was taken by Sir Donald Currie, K.C.M.G., M.P., and amongst those present were Sir Anthony Hoskings, Admiral E. S. Adeane, C.M.G., Lord Hugh Cecil, M.P., Sir Edwyn S. Dawes, K.C.M.G., Admiral H. de Kantzow, Lieut.-Colonel W. E. Despard, Admiral Sir Walter Hunt-Grubbe, K.C.B., Admiral Sir R. Vesey Hamilton, G.C.B., Captain J. J. Holdsworth, Dr. Robert Barnes, Mr. George Lidgett, J.P., Mr. P. A. Nairne, Dr. T. L. Rogers, Mr. Silas Waymouth, R.N., Captain C. A. White, Sir James Mackay, Mr. Frederick Cleeve, C.B., R.N., and their Excellencies the Hon. Thomas F. Bayard, American Ambassador, and Count Lewenhaupt, Swedish and Norwegian Minister, and the Danish Consul-General. A number of letters of apology for absence were read by the secretary, Mr. Michelli, also the annual report, which was an interesting record of work done at the four establishments which belong to the society. These are the Dreadnought Seamen's Hospital, Greenwich, the Royal Victoria and Albert Dock Hospital, the East and West India Dock Dispensary, and the Gravesend Dispensary for Seamen. The expenditure during the past year has been covered by the receipts, this result being, however, mainly due to a bequest of £2,150 from the late Mrs Eldridge. The in-patients have numbered 2,375, and the out-patients 15,918, making a total of 18,293, the highest reached in any one year by the society. By the death of Admiral the Hon. Francis Egerton last December the committee lost an able chairman and the society a friend who had been closely connected with its management since 1866.

The Chairman spoke of the formation of the society seventy-five years ago, and gave an interesting summary of its growth and work. He lamented the inadequacy of the present income and the necessity for drawing from their capital one-third of the amount needed for the year's expenses. He was of opinion that a properly arranged annual meeting, in the form of a public dinner, if inaugurated next year, might prove decidedly advantageous to the fund.

In the unavoidable absence of the Lord Mayor the adoption of the report and the election of officers for the ensuing year was moved by Count Lewenhaupt, seconded by Sir Anthony Hoskings, and carried.

A vote of thanks to various companies, associations, and other contributors was moved by the Hon. Thomas Bayard, who expressed his pleasure at the very honourable and most welcome duty which had devolved upon him. Referring to the loneliness for which sailors were sometimes pitied, he remarked that the expression "completely at sea" seemed to him to have lost the significance which formerly attached to it when it was taken to denote a very solitary condition. The Ambassador considered that isolation was now rather left on the shore by those who, escaping from contested stations, became brethren on the sea in the face of a great and mighty power. He spoke enthusiastically of the influence of the sea, which showed "man's littleness, God's power, and the necessity for befriending each other." In his own experience the pleasantest duty which he had ever performed was connected with the distribution, in his own country, of rewards to sailors for deeds of heroism on the high seas. There was a constant stream of acknowledgments passing between England and America concerning acts of bravery performed by the sailors of both countries, and friendship was strengthened by such communications. He thought it lay with all present to disprove the truth of the old saying, "out of sight, out of mind," in the case of the brave men whose lives were passed far from home and friends. He preferred to think, "lost to sight, but dear to memory." The Seamen's Hospital did not ask under what flag a man sailed, but relieved honest seamen of all nationalities, and the object of the present meeting was to strengthen the hands of those who had taken up its work.

In seconding the motion which the Ambassador had proposed, Admiral Sir Walter Hunt-Grubbe said that the excellent exposition of a sailor's life which Mr. Bayard had given left him but little to add.

The motion was carried and followed by a vote of thanks, proposed by Lord Hugh Cecil, and seconded by Mr. Frederick Cleeve, to the chairman for presiding, and to the Lord Mayor for allowing the meeting to be held at the Mansion House.

In addition to the subscriptions appearing in the annual report, Sir Donald Currie announced that he had received £605 in response to a circular which he had sent out, and to this he added his own donation of £1,000.

### VICTORIA HOSPITAL FOR CHILDREN.

The Duke of York presided over the festival dinner of the Victoria Hospital for Children, Chelsea, on Saturday evening, held at the Hotel Metropole. The guests included the Earl of Albemarle, Sir William Broadbent, Mr. Cecil Chapman, Viscount Chelsea, M.P., Mr. Alfred Farquhar (the treasurer), the Earl of Rosslyn, Lord Edmund Talbot, M.P., Sir Charles Tennant, and many others.

After the toast of "The Queen" had been proposed by the CHAIRMAN, and loyally honoured,

Viscount CHELSEA, M.P., proposed "The Health of the Prince and Princess of Wales and the rest of the Royal Family."

The Duke of YORK in replying to the toast, remarked that his father presided in 1882 at the festival dinner of the hospital, and in 1886, accompanied by his mother, opened additional buildings to the hospital, the foundation-stone of which had been laid by the Princess Louise. She had also on several occasions identified herself with the useful work of the institution, of which she is patroness, and in which she took so deep an interest. He also mentioned that the Duchess of York had since 1892 been the patroness of the Victoria Home at Margate, which is affiliated to the hospital. The Duke assured the company that it gave him very great pleasure to fill the chair that evening, not only because the charity was a most deserving one, but also because members of his family had, as he had shown, taken a personal interest in its welfare for the last twenty years. He reminded them that when the Prince of Wales presided at the dinner in 1882 Lord Cadogan and Mr. Harvey Farquhar responded for the hospital. It was a curious coincidence, and a very interesting one for him, that on this occasion he, his son, occupied the chair, Lord Chelsea, Lord Cadogan's son, had just proposed the toast, and Mr. Alfred Farquhar, son of Mr. Harvey Farquhar, was returning thanks for the toast of the hospital that night. Thus, in only fourteen years, history had repeated itself.

Later on in the evening the Duke of York in an able speech proposed "Success to the Victoria Hospital."

Colonel Sir Charles Euan Smith proposed the "Committee and the Honorary Staff," which was responded to by Mr. A. F. Dickens, Q C, and Mr. Pickering Pick.

During the evening the Secretary announced that the sum realised that night was over £3,000. This amount included £50 from the Queen, 25 guineas from the Duke of York, £25 from the Princess Louise, and £25 from the Lord-Lieutenant of Ireland.

### WEST HAM HOSPITAL.

The sixth annual general meeting of the governors and subscribers of the West Ham Hospital was held on February 26th, Alderman George Hay, J.P. (chairman of the committee), taking the chair. The report presented was an interesting one, chronicling as it did the important improvements effected in this little hospital during the past year. The great event had been the opening of the "Passmore Edwards" Wing last October, by which extension the number of beds had been increased from thirty-nine to fifty-seven. The new wing contains two wards, that on the ground floor having twelve beds for male patients, that above being devoted exclusively to children, whilst on the top floor are the nurses' comfortable quarters. The two new wards are named after Mrs. Passmore Edwards and Mr. J. R. Roberts, who have each defrayed the entire cost of furnishing the wards bearing their respective names, that furnishing, moreover, being of the most complete and thorough kind. The report strongly urged the need for additional annual subscriptions to cover the increased expenditure caused by the recent extensions. The hospital is quite free from debt, and the year closed with a balance of £1,260; but this was due to extraordinary donations and a legacy, and the amount actually derived from yearly subscriptions only covers one-fifth of the ordinary expenditure. The committee, therefore, feel that every effort must be made to find an additional £1,500, and earnestly plead for assistance in this object. The report was unanimously adopted.

